# Multifunctional Role of Astrocyte Elevated Gene-1 (AEG-1) in Cancer: Focus on Drug Resistance

**DOI:** 10.3390/cancers13081792

**Published:** 2021-04-09

**Authors:** Debashri Manna, Devanand Sarkar

**Affiliations:** Massey Cancer Center, Department of Human and Molecular Genetics, VCU Institute of Molecular Medicine (VIMM), Virginia Commonwealth University, Richmond, VA 23298, USA; debashri.manna@vcuhealth.org

**Keywords:** AEG-1, chemoresistance, RNA binding, translational regulation, protein–protein interaction

## Abstract

**Simple Summary:**

Chemotherapy is a major mode of treatment for cancers. However, cancer cells adapt to survive in stressful conditions and in many cases, they are inherently resistant to chemotherapy. Additionally, after initial response to chemotherapy, the surviving cancer cells acquire new alterations making them chemoresistant. Genes that help adapt the cancer cells to cope with stress often contribute to chemoresistance and one such gene is Astrocyte elevated gene-1 (AEG-1). AEG-1 levels are increased in all cancers studied to date and AEG-1 contributes to the development of highly aggressive, metastatic cancers. In this review, we provide a comprehensive description of the mechanism by which AEG-1 augments tumor development with special focus on its ability to regulate chemoresistance. We also discuss potential ways to inhibit AEG-1 to overcome chemoresistance.

**Abstract:**

Cancer development results from the acquisition of numerous genetic and epigenetic alterations in cancer cells themselves, as well as continuous changes in their microenvironment. The plasticity of cancer cells allows them to continuously adapt to selective pressures brought forth by exogenous environmental stresses, the internal milieu of the tumor and cancer treatment itself. Resistance to treatment, either inherent or acquired after the commencement of treatment, is a major obstacle an oncologist confronts in an endeavor to efficiently manage the disease. Resistance to chemotherapy, chemoresistance, is an important hallmark of aggressive cancers, and driver oncogene-induced signaling pathways and molecular abnormalities create the platform for chemoresistance. The oncogene Astrocyte elevated gene-1/Metadherin (AEG-1/MTDH) is overexpressed in a diverse array of cancers, and its overexpression promotes all the hallmarks of cancer, such as proliferation, invasion, metastasis, angiogenesis and chemoresistance. The present review provides a comprehensive description of the molecular mechanism by which AEG-1 promotes tumorigenesis, with a special emphasis on its ability to regulate chemoresistance.

## 1. Introduction

Until the 1960s, the mainstay of cancer treatment was surgery and radiotherapy, when it became evident that combining anticancer drugs with surgery and/or radiation treatment could prolong the survival of patients with advanced cancer, especially those having micro-metastasis [1]. The term “chemotherapy”, defined as the use of chemicals to treat diseases, was coined by the German chemist Paul Ehrlich in the early 1900s [1]. Concerted efforts by multiple groups during the first half of the 20th century allowed the development of model systems that could be efficiently used to screen for drugs having anticancer properties. These efforts, coupled with research and observations on chemicals during World War II, led to the use of nitrogen mustard to treat lymphomas and leukemias with a subsequent development of related alkylating compounds, such as chlorambucil and cyclophosphamide [2,3]. In parallel, nutritional research identified the importance of folate for cell survival, leading to the development of antifolate compounds, such as aminopterin and methotrexate, for cancer treatment [4]. Research on the potential antitumor properties of antibiotics identified actinomycin D and many other commonly used antibiotics as cancer chemotherapies, and the observation that hepatoma cells have greater uptakes of uracil compared to normal liver tissue led to the development of 5-fluorouracil (5-FU) [5,6]. These initial discoveries were followed by a multi-institutional effort pioneered by the National Cancer Institute (NCI) in the second half of the century, resulting in the development of not only broad-spectrum chemotherapy but, also, targeted therapy, including monoclonal antibodies, angiogenesis inhibitors, tyrosine kinase inhibitors (TKIs) and specific and selective inhibitors of molecules regulating tumorigenesis. Some of the commonly used chemotherapies are enumerated in Table 1.

Although many chemotherapy regimens have been adopted to treat cancer patients and milestone advancements have been made in cancer therapy development, therapeutic failure in cancer patients is a persistent and significant obstacle because of drug resistance [14].

## 2. Drug Resistance in Cancer and Its Mechanisms

Drug resistance is defined as the decrease in the efficacy and potency of a drug to generate therapeutic responses, and it is the principal limiting factor to establish a cure in cancer patients [14,15]. The resistance to chemotherapy, chemoresistance, can be either intrinsic, where tumors are resistant to chemotherapy prior to treatment, or acquired, where tumors, which are initially sensitive to chemotherapy, become unresponsive to ongoing treatment. In addition, tumors may develop a cross-resistance to a range of multiple chemotherapeutics, resulting in the development of multidrug resistance (MDR). Multiple factors contribute to the molecular mechanism of chemoresistance, and here, we highlight some of the important mechanisms.

### 2.1. Biological Factors Determining Drug Resistance

#### 2.1.1. Tumor Heterogeneity

During tumorigenesis, a diverse array of mutational processes induces genomic alterations in cancer cells, leading to the development of spatial and temporal genetic diversity [16]. The evolution of these mutational events occurs at different speeds so that some age-related mutations might be slow to develop, whereas there might be frequent gene editing by apolipoprotein B mRNA editing enzymes (APOBEC) that increase during the course of tumor development or sudden bursts of overwhelming and catastrophic events induced by genomic or chromosomal instability and chromothripsis [17,18,19]. These mutational processes, along with selective pressures, such as exogenous environmental factors, alterations in the internal environment of the tumors, e.g., hypoxia, as well as cancer treatment itself, cause the parallel and convergent development of clones that might also be segregated spatially at primary and metastatic tumors [20,21]. Tumor heterogeneity determines the responsiveness to chemotherapy, in which some clones might be sensitive to a drug while other clones in the same tumor might be resistant [22]. Chemotherapy treatment itself can create a strong selective pressure, and the plasticity of the cancer cells allow the acquisition of new resistance mutations, and alterations in signaling pathways and epigenetic gene regulations facilitating adaptations to the pressure [22]. Indeed, the treatment of low-grade gliomas with temozolomide can lead to hypermutated recurrent tumors or transform into highly aggressive glioblastoma multiforme (GBM) [23]. Advances in single-cell-based techniques, such as single-cell RNA-sequencing (scRNA-seq), can help identify multiple clonal populations in a tumor, unravel their therapeutic vulnerability and design rational and effective combinatorial treatment approaches [24].

#### 2.1.2. Overall Tumor Burden and Kinetics of Tumor Growth

The size of a tumor and the rate at which it grows determine the responsiveness to a drug [25]. The larger the tumor, or the more tumor cells in the case of liquid cancers, the greater is the likelihood of metastasis and resistance to a cure by the treatment [25,26]. A large tumor has the potential to have more mutations and, hence, more drug-resistant clones [27]. Tumors also grow at variable speeds, and it has been shown that faster-growing tumors are more sensitive to chemotherapy compared to slower, more indolent tumors [28]. At low tumor burdens, tumors grow exponentially faster and gradually reach a plateau with a slower growth rate as the tumors acquire a larger size. Upon the administration of a single dose of chemotherapy, some of the tumor cells die out, but the remaining tumor cells may resume the early phase of exponential growth, thereby reducing the effectiveness of the drug. This problem might be circumvented by dose-dense chemotherapy, in which the most effective dose of a drug is administrated over as short a time interval as possible, and the effectiveness of this approach has been demonstrated in certain cases of breast and ovarian cancers, improving the overall survival [29,30,31].

#### 2.1.3. Tumor Microenvironment (TME)

The TME consists of several types of cells, such as fibroblasts, macrophages, immune cells, endothelial cells and mesenchymal stem cells, in addition to cancer cells. The crosstalk between TME cells and cancer cells contributes to chemoresistance [32,33]. Cancer-associated fibroblasts (CAFs) secrete growth factors, e.g., hepatocyte growth factor (HGF), epidermal growth factor (EGF) and cytokines, e.g., interleukin 6 (IL-6), which activate oncogenic signaling pathways in cancer cells, resulting in chemoresistance. HGF released from CAFs activates Met in cancer cells, causing a resistance to epidermal growth factor receptor (EGFR) TKIs in lung and breast cancers [34,35]. In breast cancer, IL-6, secreted from CAFs, induced tamoxifen resistance by activating the Janus kinase/signal transducer and activator of transcription 3 (JAK/STAT3) and phosphatidylinositol 3-kinase/AKT serine/threonine kinase (PI3K/AKT) pathways, resulting in the upregulation of E3 ubiquitin ligase anaphase-promoting complex 10 activity, which targeted estrogen receptor (ER)-α degradation through the ubiquitin-proteasome pathway [36]. Another major source of IL-6 is tumor-associated macrophages (TAMs), which secrete additional cytokines, such as IL-10, IL-34 and colony-stimulating factor 1 (CSF1), all of which contribute to chemoresistance in breast, lung, colorectal, prostate and pancreatic cancers [37,38,39,40,41]. TAMs also induce extracellular matrix deposition, thereby hindering the accessibility of drugs and promoting chemoresistance in cancer cells [42]. Many solid tumors are characterized by inadequate blood flow, creating a hypoxic environment that decreases the effective exposure of the tumors to the drugs [43].

### 2.2. Factors Intrinsic to the Cancer Cells

#### 2.2.1. Drug Influx and Efflux

The accumulation of drugs inside the cells is required for a cytotoxic effect, and as such, a modulation of the influx machinery is a key factor for drug resistance. Copper transporter 1 (CTR1) is involved in cisplatin uptake and has been shown to be downregulated in ovarian cancer, resulting in cisplatin resistance [44]. In osteosarcoma, the development of methotrexate (MTX) resistance has been attributed to a decreased expression of the reduced folate carrier (RTC) [45]. In hepatocellular carcinoma (HCC), alternatively spliced variants of SLC22A1, encoding the organic cation transporter-1 (OCT1) caused decreased transport and sensitivity to sorafenib, the most common TKI used to treat advanced HCC [46].

The role of drug efflux in chemoresistance has been extensively studied in numerous cancer types [47]. ATP-binding cassette (ABC) transporters are ATPase-based membrane proteins that pump hydrophobic chemotherapeutic drugs out of cancer cells, and as such, their overexpression leads to chemoresistance. There are approximately 50 ABC transporters in the human genome, among which the most common genes overexpressed in cancers and contributing to multidrug resistance (MDR) are ABCB1 (also known as P-glycoprotein or MDR1), ABCG2 and MDR-associated proteins (MRPs). ABCB1 has a broad substrate specificity, including anthracyclines, vinca alkaloids and taxanes, and contributes to MDR in a wide range of solid and liquid cancers [48,49,50,51,52,53]. ABCG2, originally identified to provide resistance to Adriamycin in breast cancer cells, also confers imatinib resistance in HCC, gefitinib resistance in non-small cell lung cancer and doxorubicin resistance in multiple myeloma [54,55,56,57]. MDR-associated protein 1 (MRP1 or ABCC1) is involved in drug resistance in breast, lung and ovarian cancers and neuroblastoma [58]. Additional members of this family include ABCC3 functioning in breast cancer and ABCC10 (MRP7) providing paclitaxel resistance in NSCLC [59,60]. The ABC transporter-mediated chemoresistance might be overcome by using small molecule inhibitors of these transporters, such as elacridar, laniquidar or zosuquidar, or TKIs that can regulate these transporters, targeting oncogenic pathways to inhibit them or delivering chemotherapeutics using nanoparticles, thereby bypassing efflux by the transporters [61].

#### 2.2.2. Inactivation of Drugs

The effectiveness of anticancer drugs is dependent on the interaction between drugs and specific intracellular proteins. Alterations in the expression or mutation of a drug target or drug-metabolizing proteins are an important way to develop drug resistance. Aldehyde dehydrogenases (ALDHs) are a family of nicotinamide adenine dinucleotide phosphate (NADP)-dependent detoxification enzymes that play a key role in drug resistance. The human ALDH superfamily contains 19 genes, among which ALDH1A1 and ALDH3A1 have been shown to confer a resistance to a variety of chemotherapeutics, such as cyclophosphamide, doxorubicin and paclitaxel, in many different cancers [62]. The glutathione S-transferase family (GST) has a major role in the detoxification of drugs. The modulation of these GST enzymes, especially those of pi and mu classes, contribute to drug resistance in cancer cells, either directly by the detoxification of drugs or indirectly by inhibiting stress response MAP kinases, such as c-Jun N-terminal kinase (JNK) or apoptosis signal-regulating kinase (ASK1) [63,64]. Irinotecan, a topoisomerase I inhibitor used for treating colon cancer, can be inactivated by the cytochrome P-450 (CYP) family of drug metabolizing enzymes [65]. CYP subfamilies 3A and 2C play a major role in the metabolism of taxanes, such as docetaxel and paclitaxel, in the liver, as well as in solid tumors, such as breast, prostate, lung, ovarian and endometrial cancers, thus playing a role in the in-situ metabolism of these drugs and thereby affecting the intrinsic taxane susceptibility of these tumors [66]. CYP3A4 overexpression in lung and primary breast cancers has been documented to contribute to docetaxel resistance [67,68]. Cisplatin can be inactivated by the overexpression of metallothioneins (MTs), leading to cisplatin resistance in cancers [69,70].

#### 2.2.3. Modulation of DNA Damage Repair

Many of the chemotherapeutic drugs are DNA-damaging agents; as such, alterations in the DNA damage repair (DDR) process confer chemoresistance. Nucleotide Excision Repair (NER) machinery processes and removes bulky lesions, such as those created by cisplatin [71,72]. Indeed, the overexpression of NER-related gene ERCC1 (excision repair 1, endonuclease non-catalytic subunit) is associated with cisplatin resistance and negatively correlates with patient outcomes upon cisplatin treatment in non-small cell lung cancer (NSCLC) patients [73]. The DDR protein O-6-methylguanine-DNA methyltransferase (MGMT) is associated with a resistance to alkylating agents, such as nitrosoureas and temozolomide in central nervous system (CNS) tumors [74]. High levels of APEX1 (apurinic/apyrimidinic endodeoxyribonuclease 1) and PARP1 (poly(ADP-ribose) polymerase 1), involved in base exchange repair (BER), confer chemoresistance in several types of cancer [75,76]. Targeting DNA repair molecules, such as DNA polymerase β (Pol β), MGMT and N-methylpurine-DNA glycosylase (MPG), increased the sensitivity of cancer cells to alkylating chemotherapeutics [77]. The inhibition of REV3, the catalytic subunit of Pol ζ, reversed cisplatin resistance in lung adenocarcinomas [78].

#### 2.2.4. Imbalance in Apoptosis

One important chemotherapy-mediated cell death mechanism is apoptosis (programmed cell death). An imbalance in apoptosis-related proteins underlies chemoresistance development in response to conventional chemotherapeutics [79]. The overexpression of antiapoptotic protein Bcl-2 is correlated with resistance to a variety of chemotherapeutic drugs, such as 5-FU, Adriamycin, paclitaxel and mitomycin, in both liquid and solid cancers [80,81,82,83]. Another antiapoptotic protein Mcl-1 overexpression is associated with 5-FU and cisplatin resistance in oral cancer, cisplatin resistance in HCC and paclitaxel resistance in ovarian cancer [84,85,86]. Similarly, Bcl-xL overexpression conferred a resistance to cisplatin, paclitaxel, topotecan and gemcitabine in ovarian cancer [87].

#### 2.2.5. Alterations in Metabolic Pathways

Alterations in metabolic pathways are hallmarks of cancers. Compared to normal cells, cancer cells rely on aerobic glycolysis and display increased fatty acid synthesis and glutamine metabolism. Dysregulated metabolism has been demonstrated to contribute to chemoresistance in many cancers [88]. Increased glycolysis is associated with a prednisolone resistance in acute lymphoblastic leukemia [89]. An increased expression of pyruvate kinase M2 (PKM2), involved in glycolysis, serves as a biomarker for oxaliplatin resistance in colorectal and ovarian cancers, and the inhibition of PKM2 reverses this resistance [90,91,92]. The overexpression of glucose transporters is significantly correlated with chemoresistance in various cancers [93,94,95,96,97]. Taxol-resistant breast cancer cells show an increased expression of lactate dehydrogenase-A (LDH-A), an enzyme in glycolysis, and targeting LDH-A could re-sensitize these cells to Taxol [98]. In breast and pancreatic cancers, fatty acid synthase (FASN) overexpression contributes to the resistance to a wide range of chemotherapeutics [99,100]. Targeting metabolic enzymes, therefore, serves as a means to increase the chemosensitivity in multiple cancers.

### 2.3. Cancer Stem Cells

Cancer stem cells (CSCs) are a subset of cancer cells with the capacity for self-renewal, differentiation and tumorigenicity. MDR is known to be one of the key features of CSCs, which contribute to chemoresistance and recurrence. The majority of the chemotherapeutic drugs are able to inhibit tumor cells, but they are not effective towards CSCs, so drug-resistant CSC populations are enhanced following treatment, resulting in recurrence. There are several factors that regulate drug resistance in CSCs, such as, (i) pathways necessary for the maintenance of stemness, (ii) the elevated expression of ABC transporters, (iii) the overexpression of drug detoxification enzymes, such as ALDH, (iv) the inhibition of apoptotic pathways, especially those mediated by p53, (v) increased DNA damage repair ability and (vi) crosstalk with TME [101]. Enhanced CSCs are the cause of therapy resistance in several neoplasms, such as cancers of liver, breast, prostate, lung, head and neck, colon and ovary, as well as glioblastoma and leukemia [102,103,104,105,106,107,108,109,110]. The elimination of CSCs is an intensive field of research, and some of the anti-CSC strategies include (i) the inhibition of stemness pathways, such as Notch, hedgehog or Wnt pathways, using small molecule inhibitors; (ii) ALDH inhibition; (iii) the inhibition of TME cytokines, such as IL-6, using IL-6-specific antibodies and (iv) the activation of an antitumor immune response, such as by immune checkpoint inhibitors (ICI) [101].

## 3. Astrocyte-Elevated Gene-1 (AEG-1): An Oncogene Implicated in Diverse Cancers

Over the years, intense research has identified many oncogenes, tumor suppressors and signaling pathways that are potential targets for cancer therapy. Among the oncogenes, AEG-1 plays an important role in regulating tumor development and progression that includes transformation, the evasion of apoptosis, chemoresistance, angiogenesis, invasion and metastasis and negatively affects the overall patient survival in diverse human cancers [111,112]. AEG-1 was the first identified in primary human fetal astrocytes (PHFAs) by rapid subtraction hybridization (RaSH) as an HIV-, gp120- and tumor necrosis factor alpha (TNF-α)-inducible gene [113,114]. The primary localization site of AEG-1 was identified to be the endoplasmic reticulum (ER) [114]. Around the same time, AEG-1 was identified as a cell membrane protein facilitating the metastasis of breast cancer cells to the lungs and was named metadherin (MTDH) [115]. Rat/mouse AEG-1 was subsequently cloned as an ER/nuclear envelop protein and as a tight junction protein and was named the lysine-rich CEACAM-1 co-isolated protein (LYRIC) [116,117]. During the last two decades, a large body of studies has documented the elevated expression of AEG1 in a wide variety of cancers, including lung, breast, ovarian, endometrial, esophageal, gastric, hepatocellular, gallbladder, colorectal, prostate and renal cell carcinomas, glioma, neuroblastoma, melanoma, osteosarcoma and lymphomas and leukemias [111,112,118]. AEG-1 expression positively correlates with tumor progression, especially in the metastatic stage, and in vivo studies using nude mice and metastatic models with various cancer cell lines and transgenic and knockout mouse models point out that AEG-1 overexpression induces an aggressive, angiogenic and metastatic phenotype, and AEG-1 knockdown or knockout markedly hampers tumor initiation, growth and metastasis [119,120,121,122,123,124,125,126,127,128]. In addition to its role in regulating cancer, AEG-1 plays an important role in fundamental biological processes, such as inflammation, metabolism and stress response, and modulates the functions of hormones and vitamins [119,129,130,131,132,133,134,135]. AEG-1−/− mice are viable and do not show any developmental abnormality [119]. However, homozygous male AEG-1−/− mice are infertile because of a profound loss of spermatozoa as a consequence of meiotic failure [136]. In AEG-1−/− testes, an increased expression of DNA repair protein Rad18, the altered expression of Piwi-interacting RNA (piRNAs) and decreased levels of miR-16 and miR-19b, known to be reduced in the semen of infertile men, were observed, suggesting a potential role of small non-coding RNA regulation by AEG-1 in maintaining normal spermatogenesis [136].

### 3.1. Structure and Localization of AEG-1

The AEG-1 gene is located on human chromosome 8q22 and contains 12 exons and 11 introns [114]. In humans, AEG-1 is a lysine-rich highly basic protein having 582 amino acid (a.a.) residues, and the a.a. sequences are highly conserved among vertebrates [137]. Interestingly, AEG-1 is only present in vertebrates but not in lower organisms, indicating that it evolved to perform specific and specialized functions [137]. The crystal structure of the full AEG-1 protein has not been resolved, and as such, the functional domains of this protein have not been precisely defined. However, the structure of the region of AEG-1 with which it interacts with staphylococcal nuclease and Tudor domain containing 1 (SND1) has been resolved [138]. The intracellular localization of AEG-1 depends on the cell type examined and the imaging techniques employed. AEG-1 can be detectable in the cytoplasm/ER, as well as in the nucleus and nucleolus by the immunohistochemical (IHC) and immunofluorescent (IF) staining of cultured cells or tissue sections [114,117,139,140,141,142]. AEG-1 can also be found on the cell membrane in rat livers, as well as in mouse breast cancer cells [115,116].

**Figure 1:**
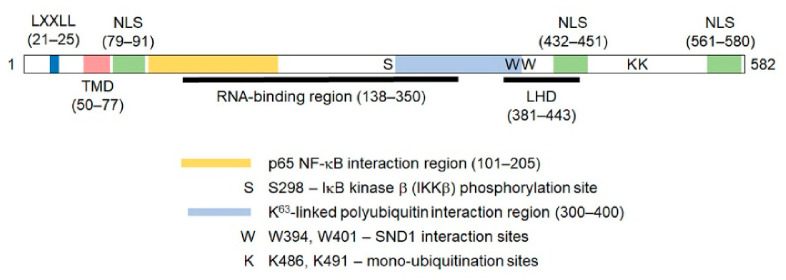
Diagram of the human Astrocyte elevated gene-1(AEG-1) protein showing the important motifs and regions mediating its function. The numbers indicate amino acid residues. The LXXLL motif allows AEG-1 to interact with retinoid X receptor (RXR) and inhibit RXR function. TMD: transmembrane domain. NLS: nuclear localization signal. LHD: lung homing domain. The K63-linked polyubiquitin interaction region mediates the interaction with the upstream molecules of the NF-B pathway, such as receptor interacting serine/threonine kinase 1 (RIP1). See text for more details.

These apparently discrepant findings can be explained by unique sequence motifs present in AEG-1 (Figure 1). AEG-1 has a transmembrane domain (TMD) between 50–77 a.a. residues that allows it to anchor onto an ER membrane, the predominant site of its localization, as well as on a cell membrane, which is mainly found in aggressive, metastatic cells [114,115,143,144]. Three nuclear localization sequences (NLS) are present in the lysine-rich regions of AEG-1 between 79–91, 432–451 and 561–580 a.a. residues, and it was shown that NLS1 and NLS3 and their flanking regions were required to target AEG-1 to the nucleus and nucleolus [117,141]. In benign human tissues, including prostate, thyroid and lung, as well as in primary mouse hepatocytes, AEG-1 is predominantly located in the nucleus, while, in cancer cells and tissues, it is located mainly in the cytoplasm [132,141]. It has been suggested that nuclear AEG-1 is a sumoylated protein that undergoes mono-ubiquitination in its NLS2 motif, facilitating its translocation out of the nucleus and increased stability in the cytoplasm [132,141]. In a later study, it was documented that K486 and K491 of AEG-1, which lie in the extended NLS2 region, undergo mono-ubiquitination, and an E3 ubiquitin ligase, TOPORS, was implicated to mediate this reaction [145]. This post-translational modification might explain why AEG-1 of a predicted 64-kD molecular weight shows bands between 70–80 kD when detected by antibodies raised against various AEG-1 immunogenic fragments. Nonetheless, the biological significance of the post-translational modification of AEG-1 in a normal body function, as well as in the pathophysiology of various diseases, remains to be elucidated. On the other hand, it has also been shown that, when stimulated by TNF-α, AEG-1 translocates to the nucleus from the cytoplasm, interacting with the p65 subunit of the nuclear factor kappa light chain enhancer of activated B cells (NF-κB) and cAMP response element binding protein (CREB)-binding protein (CBP), thereby augmenting the NF-κB transcriptional activity [139,146,147]. Additionally, a number of clinical studies have detected increasing nuclear staining for AEG-1 with the progression of cancer, although the significance of this finding has not been studied [111]. Thus, the regulation of AEG-1 localization and the mechanism of its shuttling among different intracellular compartments still requires clarification. Apart from these localization signals, a lung homing domain has been identified in mouse AEG-1, which corresponds to the 381–443 a.a. residues of human AEG-1, facilitating the adhesion of breast cancer cells to the lung endothelium [115]. AEG-1 lacks any DNA-binding domains or motifs, but it has an LXXLL motif present in its N-terminus (21–25 a.a. residues), with which AEG-1 interacts with the transcription factor retinoid X receptor (RXR) and negatively regulates its activity [132].

### 3.2. Mechanisms of Regulation of AEG-1 Expression

AEG-1 expression is regulated by diverse mechanisms. Chromosome 8q amplifications and gains are frequent events in a variety of cancers [148]. In breast cancer patients with a poor prognosis gain of chromosome 8q22, containing the AEG-1 gene was detected, and AEG-1 gene amplification was confirmed [127]. Gains of large regions of chromosome 8q with increased copy numbers of AEG-1 have also been documented in HCC [149,150]. Ha-ras activates PI3K/Akt signaling, resulting in the increasing binding of c-Myc to key E-box elements in the AEG-1 promoter, thus promoting AEG-1 transcription in transformed astrocytes [151]. It is thus expected that AEG-1 plays a pivotal role in tumorigenesis, since it is under the transcriptional control of three strong driver oncogenes, Ras, Akt and c-Myc. The overexpression and knockdown studies have established AEG-1 as a crucial nodal point in Ha-ras-mediated oncogenesis [114,151]. c-Myc is also located in chromosome 8q and is coamplified with AEG-1 in HCC patients, and a transgenic mouse with hepatocyte-specific AEG-1 and c-Myc overexpression developed highly aggressive HCC with lung metastasis, demonstrating a functional cooperation between these two molecules [122]. AEG-1 expression is induced by lipopolysaccharide (LPS) and inflammatory cytokines, such as IL-1β and TNF-α, via the activation of NF-κB, a mechanism that might contribute to AEG-1 overexpression in cancers generated as a consequence of chronic inflammation, such as HCC and gastric cancer [130,152,153]. Post-transcriptionally, AEG-1 is controlled by multiple tumor suppressor miRNAs, such as miR-375, miR-136, miR-302c, miR-466 and miR-30a-5p, which are downregulated in several cancers [154,155,156,157]. Several long noncoding RNAs (lncRNAs) have been implicated to upregulate AEG-1 by acting as a sponge for specific AEG-1-targeting microRNAs (miRNAs) [158,159,160]. In malignant glioma cells, the lncRNA human histocompatibility leukocyte antigen (HLA) complex P5 (*HCP5*) promotes malignant phenotype by upregulating Runt-related transcription factor 1 (RUNX1) via sponging miR-139, and RUNX1, in turn, upregulates AEG-1 transcription by directly binding to its promoter [161]. LINC01638 interacts with c-Myc, protecting it from the speckle-type BTB/POZ protein (SPOP)-mediated ubiquitination and degradation, with the subsequent upregulation of AEG-1 and Twist1, promoting epithelial–mesenchymal transition (EMT) in triple-negative breast cancer cells [162]. Post-translationally, mono-ubiquitination rendered an increased stabilization of cytoplasmic AEG-1 in cancer cells [141]. It was documented that cytoplasmic polyadenylation element-binding protein 1 (CPEB1) binds to AEG-1 mRNA and increases its translation in glioblastoma cells [163]. On the other hand, in HCC cells, CPEB3, which functions as a tumor suppressor, binds to the 3′-untranslated region of AEG-1 mRNA and inhibits its translation [164]. Thus, AEG-1 overexpression in cancer occurs at all levels of gene regulation.

### 3.3. Molecular Mechanism of AEG-1 Function

#### 3.3.1. Interaction with SND1

AEG-1 functions as a scaffold protein and interacts with different proteins and protein complexes, modulating their functions. The most representative protein binding with a high affinity to AEG-1 is SND1, which provides interesting insights into the mechanism of action of AEG-1 [124,165,166]. Yeast two-hybrid screening using a human liver complementary DNA (cDNA) library and coimmunoprecipitation (Co-IP), followed by mass spectrometry, identified SND1 as the protein that most strongly interacts with AEG-1 [166]. A similar strategy also identified AEG-1–SND1 interactions in breast cancer cells [165]. SND1, also known as the p100 coactivator or Tudor staphylococcal nuclease (Tudor-SN), is a multifunctional protein regulating a variety of cellular processes, such as transcription, RNA splicing and RNA metabolism [167,168,169,170]. SND1 can be found both in the nucleus and cytoplasm. It facilitates a transcription as a coactivator and mRNA splicing through interactions with the spliceosome machinery in the nucleus [171]. In the cytoplasm, it acts as a nuclease in the RNA-induced silencing complex (RISC), in which small RNAs (e.g., small inhibitory RNAs (siRNAs) or miRNAs) are complexed with ribonucleoproteins to carry out RNA interference (RNAi)-mediated gene silencing [172]. It was documented that AEG-1 interacts with SND1 in the cytoplasm, and both AEG-1 and SND1 are required for optimum RISC activity [166]. Increased RISC activity, granted by AEG-1 or SND1, was found to result in the increased degradation of tumor-suppressor mRNAs, which are targets of oncogenic miRNAs, including the mRNA of the tumor suppressor phosphatase and tensin homolog (PTEN), a target of miRNA-221, which is overexpressed in HCC [166]. Interestingly, SND1 is highly expressed in HCC, the SND1 overexpression increased and the SND1 knockdown-abrogated growth of human HCC xenografts in nude mice and a transgenic mouse with a hepatocyte-specific overexpression of SND1 (Alb/SND1) developed spontaneous and augmented diethylnitrosamine (DEN)-induced HCC [166,173]. SND1 promoted the expansion of tumor-initiating cells (TICs) in Alb/SND1 mice [173]. A selective SND1 inhibitor, 3′,5′-deoxythymidine bisphosphate (pdTp), inhibited the AEG-1-induced increased proliferation of human HCC cells and effectively reduced the tumor burden in human xenograft models of subcutaneous or orthotopic HCC [166,173]. Using a variety of mouse models, a key role of AEG-1 in the expansion of TICs in breast cancer was elucidated, facilitating metastasis, and it was documented that AEG-1 exerted its effect by interacting and stabilizing SND1 [124]. Under steady-state conditions, SND1 levels did not differ between Wild-type (WT) and AEG-1 knocked-down cells. However, upon the induction of DNA replication stress, a common type of stress during tumor development, the half-life of the SND1 protein was significantly reduced in AEG-1 knocked-down cells compared to the control, indicating that AEG-1–SND1 interactions are required for survival under stressful conditions, e.g., during tumor initiation [124]. Similarly, the overexpression of AEG-1 showed an increased stabilization of SND1 upon heat shock [138]. AEG-1 mutants, which failed to interact with SND1, lost their tumor-initiating potential [124,138]. The importance of SND1 in AEG-1-mediated oncogenesis has also been shown in clear cell renal cell carcinoma [174]. Collectively, these studies show a seminal role of AEG-1–SND1 interactions in carcinogenesis.

#### 3.3.2. Interaction with Retinoid X Receptor (RXR)

RXR is a ligand-dependent transcription factor that functions as a key regulator of cell growth, differentiation, metabolism and development [175]. RXR heterodimerizes with one-third of the 48 human nuclear receptor superfamily members, including the retinoic acid receptor (RAR), thyroid hormone receptor (TR), vitamin D receptor (VDR), Liver X Receptor (LXR), Peroxisome Proliferator-Activated Receptor (PPAR) and Farnesoid X Receptor (FXR), and regulates the corresponding ligand-dependent gene transcription. Cholesterol metabolites, fatty acid derivatives and bile acids serve as endogenous ligands for LXR, PPAR and FXR, respectively, which play an important role in regulating the lipid metabolism [175]. In the absence of ligands, RXR heterodimers interact with corepressors that maintain histones in a deacetylated state and inhibit transcription. Upon ligand binding, there is a conformational change so that the corepressors are replaced by coactivators, inducing histone acetylation and transcriptional activation. The coactivators harbor a unique LXXLL motif through which they interact with the transcription factors [176]. Interestingly, AEG-1 also harbors an LXXLL motif and yeast two-hybrid assay using the region of AEG-1 harboring the LXXLL motif-identified RXR as its interacting partner [132]. In the nucleus, the interaction of AEG-1 with RXR blocks the coactivator recruitment, thereby abrogating retinoic acid-, thyroid hormone and fatty acid-mediated gene transcription [130,131,132]. Knocking down AEG-1 markedly augmented retinoic acid-mediated killing, and this concept was used to develop and evaluate a therapeutic protocol in mouse models [132,177]. Non-thyroidal illness syndrome (NTIS), characterized by low serum 3,5,3′-triiodothyronine (T3) with normal l-thyroxine (T4) levels, is associated with malignancy, and the decreased activity of type I 5′-deiodinase (DIO1), which converts T4 to T3, contributes to NTIS [178]. T3 binds to the TR/RXR heterodimer and regulates the transcription of target genes, including DIO1. It was demonstrated that AEG-1 overexpression repressed and AEG-1 knockdown induced DIO1 expression [131]. An inverse correlation was observed between the AEG-1 and DIO1 levels in human HCC patients. Low T3 with normal T4 was observed in the sera of HCC patients and a transgenic mouse with a hepatocyte-specific overexpression of AEG-1 (Alb/AEG-1) [131]. Altogether, these observations suggested that AEG-1 might play a role in NTIS associated with HCC and other cancers.

RXR inhibitory activity allows AEG-1 to profoundly regulate the lipid metabolism. AEG-1−/− mice are significantly leaner, with prominently less body fat, compared to their WT (AEG-1+/+) littermates [129]. When fed high-fat and cholesterol diets (HFD), WT mice rapidly gained weight, while AEG-1−/− did not gain weight at all, even though their food intake was similar. AEG-1−/− mice showed decreased fat absorption from the intestines because of the increased activity of LXR and PPARα [129]. In enterocytes, the activation of LXR inhibits cholesterol absorption by downregulating cholesterol transporter Npc1l1 and upregulating the cholesterol efflux proteins Abca1, Abcg5 and Abcg8. The activation of PPARα in the enterocytes promotes the β-oxidation of absorbed fatty acids (FA), thereby downregulating fatty acid absorption into the circulation. Thus, the increased activity of LXR and PPARα in AEG-1−/− enterocytes impaired the overall fat absorption, contributing to leanness [129]. On the contrary, Alb/AEG-1 mice developed spontaneous non-alcoholic steatohepatitis (NASH), and a hepatocyte-specific AEG-1 knockout mouse (AEG-1^HEP^) was protected from HFD-induced NASH [130]. One underlying mechanism of increased steatosis in Alb/AEG-1 mice is the inhibition of PPARα-mediated FA β-oxidation, allowing the accumulation of fat in the liver [130]. Thus, AEG-1–RXR interactions have a profound implication in regulating the metabolism, as well as the functions of vitamins and hormones, especially in the liver.

#### 3.3.3. RNA-Binding Function of AEG-1

Several RNA interactome screenings identified AEG-1 as a selective ER mRNA-binding protein [179,180,181,182]. In a recent study, it was confirmed that AEG-1 is an ER-resident integral membrane RNA-binding protein (RBP) [144]. An analysis of the AEG-1 RNA interactome by the high-throughput sequencing of RNA isolated by crosslinking immunoprecipitation (HITS-CLIP) and photoactivatable ribonucleoside-enhanced crosslinking and immunoprecipitation (PAR-CLIP) methods revealed an enrichment for endomembrane organelle-encoding transcripts—most prominently, those encoding ER-resident proteins, as well as integral membrane protein-coding RNAs [144]. Secretory and cytosolic protein-encoding mRNAs were also represented in the AEG-1 RNA interactome, with the latter category enriched in genes functioning in mRNA localization, translational regulation and RNA quality control. AEG-1 does not have a consensus RNA-binding domain, and a deletion mapping analysis identified the central disordered region of AEG-1, comprised of a.a. 138–350, to bind to RNA [144]. It was shown that the overexpression of AEG-1 increases the protein levels, and not mRNA levels, of multidrug resistance gene 1 (MDR1), contributing to chemoresistance, FXII, contributing to angiogenesis, and fatty acid synthase (FASN), contributing to de novo lipogenesis, hence NASH [121,130,183]. All these three proteins are endomembranes or secreted, and it was documented that AEG-1 facilitates the association of all three mRNAs with polysomes, resulting in increased translation [121,130,183]. It should be noted that, in addition to FASN, AEG-1-bound mRNAs also code for additional fatty acid-synthesizing enzymes, and in the Gene Ontology (GO) analysis of AEG-1-bound mRNAs encoding endomembrane proteins, lipid metabolism-associated proteins were the most significant category [144]. Thus, AEG-1 promotes NASH by the translational upregulation of enzymes of de novo lipogenesis, the inhibition of PPARα-mediated FA β-oxidation and the stimulation of inflammation by activating NF-κB. A separate study also identified AEG-1 as an RBP in endometrial cancer cells by RNA immunoprecipitation, followed by a microarray [134]. However, the RNA interactome was not characterized, and it was documented that the protein levels of two AEG-1-interacting mRNAs, PDCD11 and KDM6A, were increased in AEG-1 knockdown cells, and the consequence of this observation was not studied [134]. In a follow-up study, the authors showed that, using residues 145-216, AEG-1 bound to mRNAs of FANCD2 and FANCI, two components of the Fanconi anemia complementation group that play an important role in interstrand crosslink damage induced by platinum compounds, increased their protein levels [184]. A positive correlation among the levels of AEG-1, FANCD2 and FANCI were observed in breast and endometrial cancers. Knocking out AEG-1 increased the cisplatin sensitivity in endometrial cancer cells, but a direct role of FANCD2 and FANCI in mediating this effect was not tested by overexpression/knockdown studies [184].

#### 3.3.4. Activation of the NF-κB Pathway

**Figure 2:**
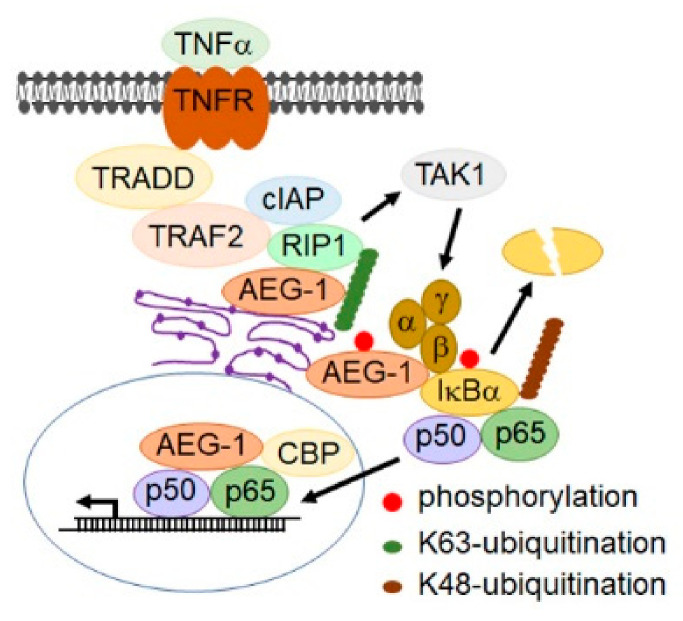
Mechanisms by which AEG-1 activates NF-B. The cartoon shows that the tumor necrosis factor (TNF)- induced signaling cascade leads to NF-B activation and the role of AEG-1 in this cascade. AEG-1, anchored on the endoplasmic reticulum (ER) membrane, associates with the upstream K63-ubiquitinated activators of NF-κB, such as RIP1 and TNF receptor associated factor 2 (TRAF2), facilitating their accumulation. AEG-1 is directly phosphorylated by IKK at serine 298, which is essential for the K48-ubiquitination of IκB followed by proteasomal degradation, facilitating the nuclear translocation of p50/p65 NF-κB. In the nucleus, AEG-1 interacts with p65 NF-κB and the CREB-binding protein (CBP) and functions as a bridging factor between NF-κB and basal transcriptional machinery, promoting NF-κB-induced transcription. IKK, and are indicated as α, β and γ in the figure.

NF-κB is a key transcriptional regulator of the inflammatory response and plays an essential role in inflammation-associated cancer [185,186]. While NF-κB induces AEG-1 expression, the first signaling pathway that was found to be activated by AEG-1 was NF-κB [139,146]. It was documented that, upon TNF-α treatment, AEG-1 translocates to the nucleus, where it interacts with the p65 subunit of NF-κB and the CREB-binding protein (CBP) and functions as a bridging factor between NF-κB and basal transcriptional machinery, promoting NF-κB-induced transcription, especially that of proinflammatory cytokines [139,146]. Subsequently, it was shown that AEG-1, anchored on the ER membrane, associates with upstream ubiquitinated activators of NF-κB, such as RIP1 and TRAF2, facilitating their accumulation and, as a consequence, NF-κB activation [143]. AEG-1 is directly phosphorylated by IκB kinase (IKK at serine 298, which is essential for inhibitory factor κB (IκB degradation and NF-κB activation [147]. Thus, AEG-1 functions in multiple steps in the NF-κB activation pathway (Figure 2), and as such, it is fundamentally required for inflammation, which has been clearly demonstrated in AEG-1-deficient mouse models [119,120].

LPS-induced NF-B activation is markedly abrogated in AEG-1−/− hepatocytes and macrophages versus WT [119]. While 16-month-old WT mice showed signs of aging-associated inflammation, no such changes were observed in the AEG-1−/− littermates, and the infiltration of macrophages was observed in aged WT livers and spleens but not in AEG-1−/− [119,129]. Indeed, AEG-1−/− mice lived longer than their WT littermates and showed a profound resistance to the DEN-induced activation of oncogenic IL-6/STAT3 signaling and development of HCC [119,129]. Communications between tumor cells and the tumor microenvironment is necessary for HCC development, and it has been shown that NF-κB activation in hepatocytes and macrophages is required for inflammation-induced HCC [187,188]. In a follow-up study, it was documented that hepatocyte-specific AEG-1 deficiency (AEG-1^HEP^) led to only an attenuation (and not complete abrogation), while myeloid-specific AEG-1 deficiency (AEG-1^MAC^) led to the complete abrogation of DEN-induced HCC, indicating that AEG-1 plays a key role in the initial macrophage activation that is crucial for hepatocyte transformation [120]. An AEG-1 deficiency made macrophages anergic, so that they did not respond to polarization stimuli, and their functional activity was markedly hampered [120]. It should be noted that AEG-1-induced inflammation has been attributed to regulate other inflammatory cancers, such as gastric cancer [133]. AEG-1 plays a seminal role in contributing to the inflammatory component of NASH, a precursor to HCC, and other inflammatory conditions, such as diabetic kidney disease, rheumatoid arthritis and HIV-1-associated neuroinflammation [130,153,189,190,191].

#### 3.3.5. Activation of PI3K/AKT Pathway

The phosphatidylinositol 3-kinase (PI3K)/Akt signaling pathway is an intracellular signal transduction pathway that promotes cell proliferation, differentiation, survival, invasion, angiogenesis, motility, metabolism and autophagy [192]. While activation of the PI3K/Akt pathway induces AEG-1, AEG-1, in turn, activates this pathway, which mediates AEG-1-mediated protection from serum starvation-induced apoptosis, as well as anoikis resistance, in multiple types of cancer [135,151,193,194]. This pathway is also important in mediating AEG-1-induced angiogenesis [126]. In less aggressive neuroblastoma cells, the overexpression of AEG-1 enhanced cell proliferation through PI3K/Akt activation and the stabilization of MYCN [195]. AKT phosphorylation by AEG-1 induced enhanced cell survival and proliferation through the suppression of forkhead box O3A (FOXO3A) activity in prostate cancer and FOXO1 in breast cancer [196,197]. Mechanistically, it was demonstrated that AEG-1 interacts with Akt2, resulting in the prolonged stabilization of Akt S474 phosphorylation and activation of downstream signaling in glioma cells [128]. It was demonstrated that AEG-1 and Akt2 expression correlated with GBM progression and reduced patient survival [128]. The AEG-1-mediated activation of PI3/Akt signaling has also been demonstrated in Alb/AEG-1 hepatocytes [121].

#### 3.3.6. Activation of the Wnt/β-Catenin Pathway

The Wnt/β-catenin pathway is an important signaling cascade for many cancers, regulating the proliferation, migration, differentiation and stemness [198]. The comparison of global gene expression changes between the control and AEG-1-overexpressed HCC cells first identified a significant modulation of the genes belonging to the Wnt/β-catenin pathway by AEG-1 [149]. AEG-1 can activate the Wnt/β-catenin pathway multiple ways: (A) AEG-1 increases the expression of lymphoid enhancer-binding factor 1 (LEF1), a transcription factor activated by Wnt signaling, and LEF1-regulated genes, such as c-Myc. (B) AEG-1 downregulates the expression of negative regulators of the Wnt pathways, like APC and C-terminal-binding protein 2 (CTBP2). (C) AEG-1 activates ERK42/44, which phosphorylates and inactivates glycogen synthase kinase 3 beta (GSK3β), resulting in the nuclear translocation of β-catenin [149]. Subsequent studies showed that AEG-1 knockdown abrogated the nuclear translocation of β-catenin, which was associated with a decrease in the EMT in HCC cells [199]. AEG-1 forms a complex with LEF1 and β-catenin, and AEG-1-mediated activation of the Wnt/β-catenin pathway facilitated the maintenance of glioma stem-like cells and their self-renewal [200]. Using Co-immunoprecipitation (co-IP) and mass spectrometry, protein arginine methyltransferase 5 (PRMT5) was identified as an interacting partner of AEG-1, and PRMT5 inhibition abrogated AEG-1-induced increases in the proliferation and migration of HCC cells [201]. It was documented that PRMT5 and β-catenin competitively bind to the same domains of AEG-1, so that AEG-1 can sequester PRMT5 in the cytoplasm, allowing β-catenin to translocate to the nucleus and regulate the gene expression [201]. The activation of the Wnt/β-catenin pathway by AEG-1-mediating EMT and metastasis has been shown in gastric, lung, cervical and tongue squamous cell carcinomas as well [202,203,204,205].

#### 3.3.7. Activation of the MAPK/ERK Pathway

The aberrant activation of the mitogen-activated protein kinase (MAPK) pathway is regularly detected in cancers and contributes to the development and progression of cancer [206]. AEG-1-mediated ERK42/44 and p38 MAPK activation was found in human HCC cells, and the inhibition of either pathway significantly inhibited AEG-1-induced cell proliferation [149]. Similar findings were also observed in Alb/AEG-1 hepatocytes with the concomitant increased activation of EGFR, an upstream activator of MAPK/ERK signaling [121,122]. A proteomic analysis of conditioned media (CM) from WT and Alb/AEG-1 hepatocytes identified the upregulation of several components of the complement pathway—most notably, Factor XII (FXII) by AEG-1, and knocking down FXII showed a decreased activation of EGFR and, consequently, MAPK/ERK [121]. These observations indicate that ligand overexpression is one mechanism by which AEG-1 activates MAPK/ERK signaling. This hypothesis is supported by the observation that AEG-1−/− primary mouse hepatocytes responded to EGF treatment, with the activation of EGFR and MAPK/ERK, to the same level compared to WT hepatocytes, indicating that AEG-1 is not required for the normal activation of MAPK/ERK, but its overexpression results in the production of aberrant ligands, such as FXII, activating the MAPK/ERK pathway [119]. The activation of MAPK/ERK results in activation of the transcription factor AP-1, a heterodimer of Fos and Jun family proteins, and it was documented that AEG-1 knockdown results in a marked inhibition of AP-1 DNA binding in prostate cancer cells [196]. In glioma cells, it was documented that AEG-1 interacts with the c-Jun/p300 complex, inducing c-Jun acetylation and increased DNA binding with a resultant enhanced expression of the target genes and increase in cell proliferation and angiogenesis both in vitro and in vivo [207]. The activation of ERK by AEG-1 induced the phosphorylation of RXR, thereby inhibiting RXR function [132]. In human retinoblastoma cells, AEG-1 knockdown significantly reduced ERK phosphorylation and showed a tumor-suppressive effect [208]. It has been reported that AEG-1 promotes non-small cell lung cancer (NSCLC) cell invasiveness by the MAPK-dependent activation of matrix metallopeptidase 7 (MMP7) [209].

#### 3.3.8. MDM2-p53 Signaling and Apoptosis

Tumor-suppressor p53 plays an important role in regulating apoptosis, and its activity is inhibited by the interaction with the E3 ubiquitin ligase transformed mouse 3T3 cell double minute 2 (MDM2), resulting in the proteasomal degradation of p53 [210]. AEG-1 protects from serum starvation-induced apoptosis, and under the serum-starved condition, the overexpression of AEG-1 activated Akt with the resultant phosphorylation of MDM2 and decreased in the levels of p53 and p21 [135]. In glioma cells, AEG-1 interacted with MDM2, preventing ubiquitination and the subsequent proteasomal degradation, resulting in an increased stabilization of the MDM2 protein [211]. The overexpression of AEG-1 protected glioma cells from apoptosis induction following MDM2 knockdown. The AEG-1 and MDM2 levels increased with the advanced stages of glioma, and high AEG-1 and MDM2 levels were associated with poor overall survival [211]. Whether this pathway is relevant in other cancers remains to be determined.

## 4. Role of AEG-1 in Cancer Drug Resistance

**Figure 3:**
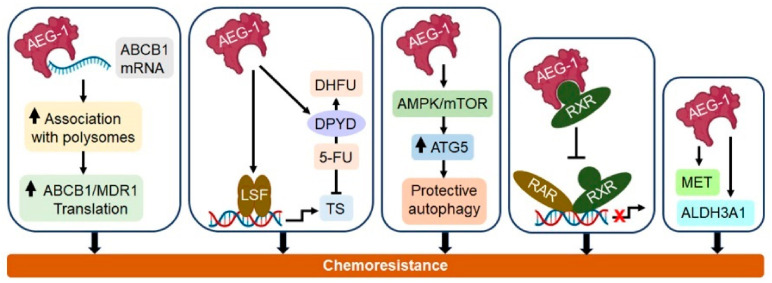
Important molecular mechanisms by which AEG-1 induces chemoresistance. AEG-1 binds to the multidrug resistance 1/ATP binding cassette subfamily B member 1 (MDR1/ABCB1) mRNA and increases its translation, thereby causing doxorubicin resistance. AEG-1 induces the transcription factor late SV40 factor (LSF), resulting in an increase in thymidylate synthase, the substrate of 5-fluorouracil (5-FU), and increases the 5-FU-metabolizing enzyme dihydropyrimidine dehydrogenase (DPYD), collectively resulting in 5-FU resistance. AEG-1 activates the AMP-activated protein kinase/mammalian target of rapamycin (AMPK/mTOR) pathway, resulting in increased ATG5 and protective autophagy, which confer a resistance to doxorubicin and 5-FU. AEG-1 interacts with retinoid X receptor (RXR), inhibiting retinoic acid receptor (RAR)/RXR-mediated gene regulation and thereby causing a resistance to retinoic acid. The AEG-1-induced expression of MET proto-oncogene, receptor tyrosine kinase (MET) and aldehyde dehydrogenase 3 family member A1 (ALDH3A1) resulted in a resistance to paclitaxel, doxorubicin and 4-hydroperoxycyclophosphamide (4-HC). See the text for more details.

AEG-1 positively regulates all hallmarks of cancer, and one major contribution of AEG-1 to the carcinogenesis process is the induction of resistance to anticancer drugs. A bioinformatics analysis of the pharmacogenomic data of the NCI-60 panel of cancer cell lines discovered a significant correlation of AEG-1 overexpression and its dual role in promoting metastasis and enhancing the chemoresistance to a broad spectrum of chemical compounds [127]. Here, we overview the current understanding of AEG-1 in the development of chemoresistance in different cancers. The important mechanisms by which AEG-1 induces chemoresistance are shown in Figure 3.

### 4.1. AEG-1 Promotes a Chemoresistance in HCC

AEG-1 is overexpressed in >90% of HCC patients [149]. The overexpression of AEG-1 increased the resistance of HCC cells to 5-FU, which is mediated by the induction of the transcription factor late SV40 Factor (LSF), resulting in an increase in the 5-FU substrate thymidylate synthase (TS) and increases in the drug-metabolizing enzyme dihydropyrimidine dehydrogenase (DPYD) [212]. In HCC cells, AEG-1 regulated the drug efflux mechanism to enhance the doxorubicin resistance [183]. AEG-1 upregulated the multidrug resistance 1/ATP binding cassette subfamily B member 1 (MDR1/ABCB1) expression, and as a result, the efflux of doxorubicin was increased, and the drug accumulation was reduced. AEG1 binds to MDR1 mRNA and increases the association of MDR1 mRNA to a polysome, resulting in augmented MDR1 protein translation without affecting the MDR1 mRNA levels [144,183]. Inhibition of the PI3K/Akt pathway could also inhibit the AEG-1-mediated polysome association of MDR1 mRNA, indicating that AEG-1 modulates the MDR1 translation in multiple ways [183]. AEG-1 overexpression increased the phosphorylation of eukaryotic translation initiation factor 4G (eIF4G) but not mTOR-sensitive eIF4E and 4E-BP, and interestingly, this activation was not blocked by the PI3K/Akt inhibitor, indicating that AEG-1 can stimulate the translational machinery in a PI3K/Akt/mTOR-independent pathway [183]. In-depth protein–protein interaction studies need to be carried out to elucidate the underlying mechanisms of this phenomenon. Nevertheless, knocking down AEG-1 significantly enhanced the sensitivity of human HCC cells to 5-FU and doxorubicin in xenograft models [183,212]. It was suggested that AEG-1 is associated with hypoxia-induced HCC chemoresistance via the PI3K/Akt/HIF-1/MDR1 pathway [213].

The tumor-suppressor miRNA miR-375 targets AEG-1. It was documented that a sorafenib treatment significantly increased miR-375 in human HCC cells, and the overexpression of miR-375 re-sensitized sorafenib-resistant HCC cells to sorafenib partially by downregulating AEG-1 [214]. It was also documented that sorafenib-resistant HCC cells showed increased levels of AEG-1 compared to their sorafenib-sensitive counterparts, suggesting that AEG-1 plays a role in acquired sorafenib resistance [214]. As a corollary, the liposome-mediated delivery of miR-375 and doxorubicin significantly inhibited human HCC xenografts by downregulating miR-375 targets, including AEG-1, as well as MDR1 [215].

Retinoic acid (RA) and its analogs are routine cancer therapeutics for leukemia, and they have been evaluated in Phase II/III clinical trials for the prevention and treatment of HCC, although they did not progress further [216]. RA mediates its effect by retinoic acid receptor (RAR)/RXR, and the overexpression of AEG-1 inhibits RAR/RXR activity, thereby inducing a resistance to RA [132]. The delivery of AEG-1 siRNA by hepatocyte-targeted nanoparticles in combination with all-trans retinoic acid (ATRA) resulted in the profound inhibition of orthotopic xenografts of human HCC cells compared to either agent alone [177]. These findings indicate that a combination with AEG-1 inhibition might establish ATRA or other RA analogs again as a viable treatment option for HCC.

### 4.2. Breast Cancer Chemoresistance and AEG-1

Worldwide, breast cancer is the most common malignant tumor observed in women [217]. AEG-1 overexpression by 8q22 genomic gain is frequently observed in poor-prognosis breast cancer patients and plays an important role in breast cancer chemoresistance and metastasis [127]. It was documented that AEG-1 conferred a resistance to broad-spectrum chemotherapeutics, such as paclitaxel, doxorubicin and 4-HC, by upregulating aldehyde dehydrogenase 3 family, member A1 (ALDH3A1) and MET [127]. Estrogen-independent growth promotes resistance to one of the selective estrogen receptor modulators (SERMs), tamoxifen, which is clinically the first line of treatment for patients with ER-positive breast cancer. AEG-1 overexpression in MCF-7 cells enhanced estrogen-independent growth and tamoxifen resistance by reducing the expression of PTEN and upregulating AKT and BCl-2, thereby inhibiting tamoxifen-induced apoptosis [218]. In breast cancer, AEG-1 promoted CSC expansion by increasing the transcription of TWIST1, a transcription factor critical for metastasis and stemness [219]. AEG-1 interacted with the histone acetyltransferase CBP, preventing the ubiquitin-mediated degradation of CBP and allowing it to promote TWIST1 transcription. A positive correlation between the AEG-1 and TWIST1 levels was observed in breast cancer clinical samples [219]. It was also documented that the ectopic expression of AEG-1 increased the expression of ABCB1 and ABCG2 in the CSCs and augmented the paclitaxel resistance [219]. The AEG-1 expression levels in multiple breast cancer cell lines showed negative associations with doxorubicin sensitivity [220]. Similarly, the nanoparticle-mediated delivery of AEG-1 siRNA effectively downregulated the AEG-1 expression and sensitized MCF-7 cells to paclitaxel [221]. A high expression of AEG-1 correlated with a poor prognosis of locally advanced human epidermal growth factor receptor 2 (HER-2) positive breast cancer patients after treatment with neoadjuvant chemotherapy and trastuzumab, suggesting a potential role of AEG-1 in conferring trastuzumab resistance [222].

### 4.3. Chemoresistance in Glioma

AEG-1 is highly upregulated in glioma and confers chemoresistance in multiple ways [223]. The overexpression of AEG-1-induced protective autophagy in multiple cell types by activating the AMPK/mTOR pathway led to increased expression of the autophagy regulator ATG5, and knocking down AEG-1 in glioma cells significantly increased their sensitivity to doxorubicin [224]. AEG-1 interacts with Akt2 to prolong Akt activation, which is required for the growth and survival of glioma, and the inhibition of the AEG-1-Akt interaction rendered glioma cells as more sensitive to temozolomide (TMZ) [128]. Similarly, the inhibition of AEG-1 by miR-136 enhanced the TMZ sensitivity in U251 glioma cells [225].

### 4.4. Endometrial and Cervical Cancer

AEG-1 is highly expressed in advanced endometrial cancer cells, and knocking down AEG-1 in endometrial cancer cells increased the sensitivity to tumor necrosis factor-α-related apoptosis-inducing ligand (TRAIL) and histone deacetylase (HDAC) inhibitor LBH589 [226]. In AEG-1-depleted cells, the inhibition of phosphoinositide-dependent protein kinase 1 (PDK1) and AKT phosphorylation, along with increased Bim expression and XIAP degradation, correlated with an enhanced sensitivity to these drugs. Additionally, the gene expression analysis suggested a potential role of calbindin 1 and galectin-1 in contributing to AEG-1-mediated therapeutic resistance [226]. AEG-1 positively regulates the expression of FANCD2 and FANCI, which regulates DNA damage by platinum compounds, and knocking out AEG-1 in endometrial cancer cells significantly increased their sensitivity to cisplatin [184]. Pristimerin, a traditional Chinese medicine, significantly decreased the AEG-1, FANCD2 and FANCI levels in endometrial cancer cells and restored their sensitivity to cisplatin in an in vivo xenograft model [184]. In multiple cervical cancer cell lines, the overexpression of AEG-1 induced autophagy and activated the ERK/NF-κB pathway, conferring a resistance to cisplatin [227]. The knockdown of AEG-1 was also shown to abrogate the EMT and increase the sensitivity to cisplatin and paclitaxel in cervical cancer cells [228]. The E6/E7 proteins of human papillomavirus 16/18 downregulated miR-375 in cervical cancer cells, resulting in the upregulation of AEG-1 and resistance to 5-FU [229].

### 4.5. Ovarian Cancer

Samples from 101 patients with stage II-IV ovarian serous carcinoma were divided into either cisplatin-resistant or cisplatin-sensitive groups based on the information regarding a relapse or complete remission following six cycles of cisplatin chemotherapy [230]. A high AEG-1 expression was detected in all cisplatin-resistant patients and correlated with a shorter overall survival compared to the cisplatin-sensitive patients [230]. As a corollary, in epithelial ovarian carcinoma, a high AEG-1 expression was significantly associated with neoadjuvant chemotherapy-resistant patients versus -sensitive patients, predicting a poor prognosis [231]. The traditional Chinese herbal formula Guizhi Fuling Wan (GFW) treatment restored cisplatin sensitivity in cisplatin-resistant SKOV3 ovarian cancer cells by inhibiting AEG-1, increasing PTEN and modulating the AEG-1/PTEN interaction [232].

### 4.6. Lung Cancer

The elevated expression of AEG-1 is associated with non-small cell lung cancer (NSCLC) progression, leading to a poor clinical outcome [233]. NSCLC is treated with the antimetabolite drug pemetrexed, and higher thymidylate synthase (TS) levels are associated with pemetrexed resistance. Knocking down AEG-1 in the pemetrexed-resistant lung cancer cell line downregulated the TS levels and decreased the half-maximal inhibitory concentration (IC_50_) value of pemetrexed [234]. An analysis of the repeated biopsy samples documented increased the levels of TS and AEG-1 with the progression of the disease [234]. The RNA immunoprecipitation analysis identified the interactions among circMTDH.4, miR-630 and AEG-1 in NSCLC, with circMTDH.4 regulating the AEG-1 expression by sponging miR-630 [235]. The overexpression and knockdown studies identified a role of this axis in conferring the chemoresistance/radioresistance in NCI-H1650 and A549 cells [235]. 5-FU-resistant A549 cells displayed the decreased expression of miR-124-5p and increased levels of its target gene, AEG-1 [236]. The overexpression of AEG-1 reversed the ability of miR-124-5p to increase the sensitivity of these cells to 5-FU [236]. Collectively, these studies documented that AEG-1 confers both an inherent and acquired chemoresistance to NSCLC cells.

### 4.7. Prostate Cancer

An interesting study in prostate cancer used AEG-1 as an antigen to mount an antitumor immune response. Mice bearing orthotopic prostate cancers were immunized with AEG-1 antigen carried by attenuated *Salmonella* [237]. This approach induced a CD8+ T cell-mediated immune response inhibiting tumor growth and metastasis and increased the chemosensitivity to paclitaxel with a prolongation of the survival time [237]. The inhibition of AEG-1 in prostate cancer cells increased the sensitivity to cisplatin through modulating the PI3K/AKT pathway [238].

### 4.8. Miscellaneous Cancers

AEG-1-mediated chemoresistance has been shown in additional cancers. Similar to glioma, AEG-1-induced autophagy, AMPK/ATG5 signaling and an increase in MDR1 have been implicated to confer 5-FU resistance in gastric cancer [239]. AEG-1-induced autophagy was also shown to play a role in mediating Adriamycin resistance in T-cell Non-Hodgkin’s lymphoma [240]. The knockdown of AEG-1 in neuroblastoma cells induced cell cycle arrest in the G0/G1 phase and increased the sensitivity to cisplatin and doxorubicin [241]. The expression of miR-let-7b and miR-let-7c were downregulated in mucosal melanoma, and it was shown that these miRNAs target AEG-1, and their overexpression increased the sensitivity to paclitaxel, which could be reversed by AEG-1 overexpression [242]. In osteosarcoma cells, AEG-1 induced endothelin-1 (ET-1) via PI3K/AKT pathway activation [243]. Knocking down AEG-1 increased cisplatin-mediated killing, which could be rescued by ET-1 treatment, and conversely, the inhibition of ET-1 signaling further potentiated this effect, suggesting a potential role of ET-1 in mediating AEG-1-induced chemoresistance [243]. Table 2 shows a snapshot of AEG-1-mediated drug resistance in different cancers.

## 5. Conclusions

From the above discussion, it is clear that AEG-1 plays a key role in regulating the chemoresistance, and it is a bona fide target to overcome chemoresistance and increase chemosensitivity. The major obstacle to this strategy is a lack of availability of a small molecule inhibitor of AEG-1. There are several obstacles to developing such an inhibitor. First, AEG-1 mediates its function by interacting with multiple proteins using different regions, so that the inhibition of one interaction may not be able to inhibit other interactions crucial to its function, e.g., the inhibition of the AEG-1/Akt2 interaction may block the AEG-1-induced activation of the PI3K/Akt pathway but may not affect the AEG-1-induced activation of NF-B or inhibition of RXR. Second, the crystal structure of full AEG-1 protein has not yet been resolved. Transmembrane proteins are difficult to crystalize, and efforts to generate a sufficient amount of AEG-1 protein for crystallization has been daunting because of the inherent stickiness of the protein (DS unpublished observation). The resolution of the AEG-1/SND1 interaction regions identified two key tryptophan (W) residues at a.a. 394 and 401 of AEG-1 mediating, this interaction thus paving the way for the generation of potential peptidomimetics, as well as small-molecule inhibitors to block this interaction [138]. In a recent study, AEG-1-interacting domains of SND1 were used as bait in a phage display screening to identify a 12-a.a. peptide that could disrupt the AEG-1–SND1 interaction in vivo, induce SND1 degradation and inhibit the growth of human breast cancer xenografts [244]. Whether this strategy works in other cancers and overcomes the chemoresistance remains to be determined.

RNA interference (RNAi) is another way by which AEG-1 can be targeted, because this approach has shown efficacy in preclinical models and phase I clinical trials for other oncogenes in cancers such as HCC [245,246,247]. Indeed, nanoparticle-mediated targeted delivery for the in vivo inhibition of AEG-1 is a clinically relevant strategy in the context of HCC, as the payload delivery after intravenous administration is the highest for liver compared to other organs. Hepatocyte-targeted nanoplexes were developed by conjugating polyamidoamine (PAMAM) dendrimers with polyethylene glycol (PEG) and galactose lactobionic acid (PAMAM-PEG-Gal), which were complexed with AEG-1 siRNA (PAMAM-AEG-1si) [177]. AEG-1 overexpression induces a resistance to ATRA [132]. In a nude mice orthotopic human HCC xenograft assay, it was documented that ATRA alone had no effect on tumor growths, PAMAM-AEG-1si alone significantly inhibited tumor growths and the combination of PAMAM-AEG-1si and ATRA completely eliminated the tumors without exerting any toxicity [177]. In a separate study, it was documented that PAMAM-AEG-1si could effectively protect C57BL/6 mice from HFD-induced NASH development [130]. Thus, PAMAM-AEG-1si could be an HCC therapeutic strategy for HCC, as well as an HCC preventive strategy by inhibiting NASH. AEG-1 knockdown markedly augmented the anti-HCC activity of Dox and 5-FU, indicating that PAMAM-AEG-1si can be combined with standard chemotherapy or TKIs [183,212]. AEG-1 plays a profound role in regulating macrophage functions and inflammation, and as such, PAMAM-AEG-1si can also be combined with anti-inflammatory strategies and immunotherapy. In-depth studies using endogenous mouse models of HCC need to be performed to evaluate these strategies for their potential transition to clinics. A recent study described the therapeutic efficacy of locked nucleic acid (LNA)-modified AEG-1 antisense oligonucleotide (ASO) in inhibiting the primary tumor growth and attenuating the metastasis of syngeneic breast, colorectal and lung tumors in C57BL/6 mice, further establishing the efficacy of a gene-based strategy for inhibiting AEG-1 [125].

As yet, most studies have focused on the role of AEG-1, contributing to the inherent resistance of cancer cells to standard chemotherapy. A few studies have documented AEG-1 overexpression in experimentally generated drug-resistant clones compared to the parental drug-sensitive cells, and clinical correlation studies between drug-sensitive and drug-resistance patients have demonstrated a higher AEG-1 expression in drug-resistant patients [214,230,231,236]. However, in-depth clinical studies need to be performed to analyze the AEG-1 expression during disease progression and in response to treatment, especially following a relapse and recurrence after a course of treatment to determine whether AEG-1 alterations/overexpression contribute to the acquired resistance. Only a few studies have explored the role of AEG-1 in radioresistance, and the underlying molecular mechanism is mostly unexplored; thus, there is a vast scope of research in this field [235,248,249]. The cancer treatment strategy is moving more and more away from broad-spectrum chemotherapeutics and toward targeted therapy and immunotherapy. A handful of studies have explored the involvement of AEG-1 in mediating the resistance to these modalities, such as sorafenib, trastuzumab or HDAC inhibitors, and as such, there is a large black box that needs to be interrogated [128,214]. With the unraveling of a key role of AEG-1 in the macrophage function, its potential role in modulating immunotherapy needs to be explored as well. Comprehensive, in-depth and detailed mechanistic and translational studies using preclinical and clinical models will further establish the importance of AEG-1 in the diagnosis, prognosis and treatment of cancers.

## Figures and Tables

**Table 1:** Commonly used chemotherapeutics and their mechanisms of action.

Type of Drug	Mechanism of Action	Examples	Reference
Alkylating agents	DNA damage	Platinum compounds (cisplatin, carboplatin, oxaliplatin), cyclophosphamide, darabazine, chlorambucil, temozolomide	[7]
Nitrosoureas	DNA damaging agents crossing the blood–brain barrier	Streptozocin, lumustine	[8]
Anti-metabolites	Interfere with DNA and RNA by acting as a substitute for normal building blocks of DNA and RNA	Azacitidine, 5-FU, 6-mercaptopurine, decitabine, gemcitabine, hydroxyurea, methotrexate, pemetrexed, thioguanine	[9]
Antibiotics: anthracyclines	Interfere with enzymes that copy DNA	Daunorubicin, doxorubicin (Adriamycin)	[10]
Antibiotics: non-anthracyclines	Diverse mechanisms, such as DNA intercalation and DNA strand break	Bleomycin, mitomycin-C, mitoxantrone	[11]
Topoisomerase inhibitors	Plant alkaloids that interfere with topoisomerases required for DNA strand separation	Topoisomerase I inhibitors (camptothecins): irinotecan, topotecanTopoisomerase II inhibitors: etoposide, mitoxantrone	[12]
Mitosis inhibitors	Plant alkaloids that inhibit cell division	Taxanes: docetaxel, paclitaxelVinca alkaloids: vinblastine, vincristine, vinorelbine	[13]

**Table 2:** AEG-1-mediated drug resistance in different cancers.

Cancer Site	Drug	Study Material and Type of Study	Targets/Pathways	References
HCC	Doxorubicin	In vitro and nude mice xenograft studies using QGY-7703 and AEG-1 overexpressing clones of HepG3 cells.	AEG-1 binds to MDR1/ABCB1 mRNA and increases its translation. AEG-1 also inhibits ubiquitination and proteasome-mediated degradation of MDR1.	[144,183]
	5-FU	In vitro and nude mice xenograft studies using QGY-7703 and AEG-1 overexpressing clones of HepG3 cells.	AEG-1 induces expression of LSF which transcriptionally regulates 5-FU substrate thymidylate synthase (TS). AEG-1 also induces DPYD which catalyzes the initial and rate-limiting steps of 5-FU catabolism	[212]
	Sorafenib	In vitro and nude mice xenograft studies using sorafenib-resistant Hep3B and HepG2 cells.	Sorafenib induces miR-375 which targets AEG-1	[214]
	Retinoic Acid	In vitro and nude mice xenograft studies using QGY-7703 and AEG-1 overexpressing clones of HepG3 cells. Primary hepatocytes from Alb/AEG-1 and AEG-1-/- mice.	AEG-1 interacts with RXR and prevents co-activator recruitment thus inhibiting RAR/RXR function. AEG-1 also traps RXR in the cytoplasm.	[132,177]
Breast cancer	Paclitaxel, doxorubicin and 4-HC	In vitro and nude mice xenograft studies using LM-2 (a MDA-MB-231 subline) and SCP28 cells. Tumor specimens from breast cancer patients.	AEG-1 induces expression of ALDH3A1 and MET	[127]
	Tamoxifen	In vitro studies using MCF-7 cells.	AEG-1 reduces PTEN and upregulated AKT and BCL2	[218]
	Paclitaxel	In vitro studies using cancer stem cells (CSCs) obtained from MDA-MB-231 and MCF-7 cells. Tumor samples from breast cancer patients.	AEG-1 interacts with CBP which promotes histone H3 acetylation on the twist family bHLH transcription factor 1 (TWIST1) promoter and induces TWIST1 expression. TWIST1 contributes to development of CSCs which are resistant to paclitaxel.	[219]
	Neoadjuvant chemotherapy and trastuzumab	Breast cancer patients treated with trastuzumab and neoadjuvant chemotherapy	Not identified	[222]
Glioma	Doxorubicin	In vitro studies using immortalized primary human fetal astrocytes	AEG-1 promotes AMPK/mTOR/ATG5-induced protective autophagy	[224]
	Temozolomide	In vitro and nude mice xenograft studies using U87, U251 and primary human glioma cells (VG2, VG4 and VG6)	AEG-1-Akt2 interaction stabilized phosphorylated Akt2 promoting survival	[128]
		In vitro studies using U251 cells	miR-136 targets AEG-1 and increases sensitivity to temozolomide	[225]
Endometrial cancer	TRAIL and HDAC inhibitors	In vitro studies using RL95, AN3CA, KLE, Ishikawa, Hec50co and ECC1 cells	AEG-1 induces expression of Calbindin 1 and galectin-1 and activates AKT	[226]
	Cisplatin	In vitro studies using Hec50 and KLE cells	AEG-1 induced expression of fanconi anemia, complementation group D2 and I (FANCD2 and FANCI)	[184]
Cervical cancer	Cisplatin	In vitro studies using SiHa, HeLa, CaSki, and C33A cells	AEG-1-induced autophagy and increased activation of ERK/NF-κB	[227]
	5-FU	In vitro studies using HeLa (HPV-18), SiHa, CaSki (HPV-16), and C33A (HPV-negative)	Downregulation of miR-375 which targets AEG-1	[229]
Ovarian cancer	Cisplatin	Patients with stages III–IV ovarian serous carcinoma	Not identified	[230]
	Neoadjuvant chemotherapy	Epithelial ovarian carcinoma patients who underwent debulking surgery after neoadjuvant chemotherapy	Not identified	[231]
Lung cancer	Pemetrexed	In vitro studies using A549, H157, H520, H292, CL1-0, CL1-5, PC-9 and H1975 cells	AEG-1 induced expression of Thymidylate synthase	[234]
	5-FU, cisplatin, radiation	In vitro and in vivo xenograft studies using NCI-H1650 and A549 cells	circMTDH.4/miR-630/AEG-1 axis was identified to confer chemo- and radioresistnace	[235]
	5-FU	In vitro studies using A549, H1299 cells and A549/5-FU clones.	Increased miR-124-5p was associated with AEG-1 downregulation and increased chemosensitivity	[236]
Prostate cancer	Cisplatin	In vitro studies using PC3, DU145 and LNCap	AEG-1 activates PI3K/AKT pathway	[238]
	Paclitaxel	Orthotopic implantation of mouse prostate cancer cell RM-1 in C57BL/6 mice	AEG-1 vaccine enhanced sensitivity to paclitaxel	[237]
Gastric cancer	5-FU	In vitro studies using GC AGS, SGC7901, BGC823, HGC803, and MKN45 cells. Tumor samples from gastric cancer patients	AEG-1 promoted AMPK/ATG5-induced autophagy	[239]
T-cell Non-Hodgkin’s lymphoma	Adriamycin	In vitro studies using Hut-78 and Jurkat cells. T-NHL tissues.	AEG-1-induced autophagy	[240]
Melanoma	Paclitaxel	In vitro studies using HMVII and GAK cells. Mucosal melanoma patient tissues.	AEG-1 reversed sensitivity conferred by miR-let-7b/miR-let-7c to paclitaxel	[242]
Osteosarcoma	Cisplatin	In vitro studies using Saos-2 and MG-63 cells	AEG-1 induces endothelin-1	[243]
